# The risk of immune-mediated inflammatory diseases following exposure to childhood maltreatment: A retrospective cohort study using UK primary care data

**DOI:** 10.1016/j.heliyon.2024.e40493

**Published:** 2024-11-16

**Authors:** Liam Snook, Sonica Minhas, Vrinda Nadda, Ben Hammond, Krishna M. Gokhale, Julie Taylor, Caroline Bradbury-Jones, Siddhartha Bandyopadhyay, Krishnarajah Nirantharakumar, Nicola J. Adderley, Joht Singh Chandan

**Affiliations:** a Department of Applied Health Sciences, College of Medical and Dental Sciences, University of Birmingham, B152TT, UK; b Barts and the London School of Medicine and Dentistry, Queen Mary University of London, E12AD, UK; c School of Nursing and Midwifery, College of Medical and Dental Sciences, University of Birmingham, B152TT, UK; d Department for Economics, University of Birmingham, B152TT, UK; e National Institute for Health and Care Research (NIHR) Birmingham Biomedical Research Centre, UK; f Birmingham Health Partners, UK

**Keywords:** Childhood maltreatment, Autoimmune disease, Epidemiology, Retrospective cohort study

## Abstract

**Background:**

As a global public health issue, childhood maltreatment is associated with significant morbidity and mortality. We aimed to investigate the association between childhood maltreatment and immune-mediated inflammatory disorders (IMIDs).

**Methods:**

We conducted a retrospective matched open cohort study using a UK primary care database between January 1, 1995 and January 31, 2021. Clinical codes were used to identify patients exposed to childhood maltreatment who were matched by general practice (GP), age, and sex to up to four unexposed patients. Cox regression analysis was used to evaluate the risk of developing IMIDs (inflammatory bowel disease, coeliac disease, rheumatoid arthritis, psoriasis, multiple sclerosis, systemic lupus erythematosus) during follow-up in the exposed versus unexposed groups.

**Results:**

256,130 exposed patients were matched to 712,478 unexposed patients. Those exposed to childhood maltreatment were 1) at an increased risk of developing Rheumatoid arthritis (aHR 1·39; 95 % CI 1·12-1·74) and Psoriasis (aHR 1·16; 95 % CI 1·10-1·23), 2) not statistically significantly at risk of developing inflammatory bowel disease (aHR 0·87; 95 % CI 0·75-1·00), multiple sclerosis (aHR 1·07; 95 % CI 0·77-1·49) and systemic lupus erythematosus (aHR 1·28; 95 % CI 0·89-1·85) and 3) at a reduced risk of coeliac disease (aHR 0·74; 95 % CI 0·62-0·88) compared to the unexposed group.

**Interpretations:**

Childhood maltreatment is estimated to affect one in three children globally; therefore, an increased risk of developing rheumatoid arthritis and psoriasis represents a substantial contribution to the burden of IMIDs. Implementation of broad public health approaches to prevent and detect childhood maltreatment and its negative downstream consequences, such as, IMID development, is essential.

## 1 Introduction

Childhood maltreatment, a violation of human rights, is defined as any form of physical, sexual, or emotional abuse, including neglect [1,2]. It is associated with substantial morbidity and mortality, hence is a recognised global public health issue [[3], [4], [5], [6], [7], [8], [9]]. The prevalence of childhood maltreatment is disproportionately distributed, with boys more likely to experience physical abuse and girls sexual abuse, whilst neglect is more common in areas of higher deprivation [10,11]. The retrospective nature of case ascertainment in cross-sectional studies makes it difficult to accurately determine the exact prevalence of childhood maltreatment [12]. However, studies derived from healthcare records describe an increasing prevalence of childhood maltreatment, which may be due to improved recognition and reporting or an increasing population [13,14].

Immune-mediated inflammatory disorders (IMIDs) are a group of common chronic and diverse autoimmune inflammatory conditions that are highly disabling with currently no curative therapies [15]. They are characterised by shared changes in common immune pathways causing dysregulation. Yet, present unique differences in other pathways that define their own clinical picture: phenotype, age and sex distribution, and tissue localisation [15]. Broadly, IMIDs can be categorised based on the presence of autoantibodies (seropositive, e.g., coeliac disease, rheumatoid arthritis), or the lack thereof (seronegative, e.g., Crohn's disease, psoriasis) [16]. Despite the distinct pathogenic differences between these categories, genome-wide association studies have shown genetic overlap across all these phenotypes, providing evidence for shared pathways [17]. Importantly, the genetic overlap indicates having one IMID increases risk of developing another [15,18]. The development of disease-modifying drugs has increased patient survival. IMIDs have an estimated IR of 80 per 100,000 person-years, but this is expected to rise, highlighting the importance of preventing future cases [19].

It is poorly understood how childhood maltreatment could lead to the development of IMIDs. There is an increasing evidence base that hypothesises childhood maltreatment could induce an abnormal inflammatory response that exacerbates the development of IMIDs [20,21]. Those exposed to childhood maltreatment are also more likely to exhibit unhealthy behaviours, such as smoking and drinking excess alcohol [22,23], which can contribute to the pro-inflammatory environment, increasing the risk of IMID development [24,25]. Other factors associated to immune dysregulation that could explain this increased risk include obesity, and mental disorders [26,27].

Previous literature supports an association between childhood maltreatment exposure and development of specific IMIDs. The most recent meta-analysis was conducted in 2009 which evaluated exposure to childhood maltreatment and the onset of IMIDs. Eight of the included 24 studies (n = 48,801) measured the effect sizes of developing an IMID after childhood maltreatment (d = 0.23 [95 % CI: 0.19–0.27]), concluding an increased risk in those abused [21]. Further, Dube et al., conducted a retrospective cohort study of 15,357 adults, evaluated the risk of developing IMIDs following childhood maltreatment, with the risk of hospitalisation for one of 21 autoimmune diseases (including systemic lupus erythematosus, rheumatoid arthritis, multiple sclerosis, inflammatory bowel disease, psoriasis, and coeliac disease) being higher amongst adults with with 2 or ≥3 adverse childhood exposures (ACEs) compared to those with no ACEs [20]. However, this study is limited in its identification of autoimmune disease outcomes through hospitalisations and not outpatient data, as well as the risk of recall bias due to its design. On the whole, due to temporal limitations, the influence of recall bias, and issues with generalisability due to geographical structural differences, the relationship between childhood maltreatment and IMIDs has not been established conclusively in the UK [28].

## 2 Methods

### 2.1 Study design, setting, and population

In light of research gaps we aimed to conduct a large retrospective open cohort study, using UK primary care data, to assess the risk of developing six IMIDs (inflammatory bowel disease (IBD), coeliac disease, rheumatoid arthritis (RhA), psoriasis, multiple sclerosis (MS), systemic lupus erythematosus (SLE)) in those exposed to childhood maltreatment compared to those unexposed. These have been selected as outcomes due to their citations in literature as the most common and detrimental on quality of life, thus having clinical significance [28].

Study data for this retrospective, matched open cohort study was obtained from the IQVIA Medical Research Database (IMRD) UK database. IMRD includes data from The Health Improvement Network (THIN), which is a Cegedim database containing longitudinal, de-identified, primary care electronic medical records. IMRD is a primary care database that represents the prevalent comorbidities and demographic composition of the UK population due to its large size [29]. The January 1, 1995 to the January 31, 2021 was set as the study period.

IMRD-UK comprises of data from 832 general practices (GPs) that use the Vision electronic medical records system. The IMRD-UK database contains records more than 16 million patients in the UK, actively covering 6 % of the UK population [29]. Although the regional coverage of the dataset ranges from 4.2 % to 8.9 % in the north and south of England respectively, the dataset has been shown to be representative of the general population in terms of demographics and the prevalence of key co-morbidities [14]. Symptoms, examinations, and diagnoses recorded in IMRD-UK are recorded using a hierarchical clinical coding system, called Read codes [30].

Practices became eligible for the study at the latest of the following: one year post-installation of the practice electronic medical records system (to allow enough time for systems to transfer patient data), or the date the practice reached acceptable mortality recording [31]. This minimised the under-reporting of outcomes and ensured accurate recording of clinical details in electronic records. Acceptable mortality recording is reached when a practice reports mortality rates similar to their populations expected rate, which is outlined by the Office for National Statistics. This measures how accurately practices record death, providing insight into the quality of a practice's imputation into electronic medical records [32]. The ‘Data extraction for epidemiological research (DExtER)’ tool was used to facilitate the extraction, transformation and loading of data [33].

### 2.2 Exposure and outcome definitions

Exposure to childhood maltreatment was determined if the electronic medical record contained a Read code (appendix 1) describing both GP-recorded childhood maltreatment or maltreatment-related concerns. Including both reduced the risk of under-reporting. Codes related to GP-recorded childhood maltreatment comprised of exposure to abuse (physical, sexual, or emotional), neglect, and domestic abuse through observed injuries, self-report, parent-report or third-person report. Maltreatment-related codes included concerns such as social services involvement, a child considered to be at risk, and suspected or possible maltreatment and neglect. There was no age restriction to study entry, both adults and children were included in this study. Patients included in the exposed group of this study had an exposure to maltreatment or a maltreatment-related concern recorded by a GP before the age of 18 years. The coding of childhood maltreatment in primary care has been improved through past work [34,35]. Previous literature that used the THIN database to investigate childhood maltreatment have been adapted to select the maltreatment and related code lists for this study [14]. The outcome of interest was also defined by Read codes (appendix 1); IMID (IBD, coeliac disease, RhA, psoriasis, MS, and SLE). There is no evidence to suggest the recording of IMIDs is validated in THIN, however this group of IMIDs have been selected as they have been grouped in previous literature and been explored in GP records. It was anticipated that RhA would be well-coded, as it is included in the Quality Outcomes Framework to mandate GP coding as a performance indicator [36]. General practitioners and public health clinicians assisted to curate these code lists, and is supported by wider literature [37].

Random matching was employed to match each exposed patient to up to four unexposed patients (those with no recorded Read code exposure to childhood maltreatment). They were matched for general practice, sex, age ( ± one year), Townsend deprivation quintile, and index date.

### 2.3 Follow-up period

The date when a participant starts contributing person-years of follow-up and is deemed to be eligible for the study is known as the index date. For incident cases, the index date for exposed patients was the date of first Read code relating to childhood maltreatment within the study eligibility period. For prevalent cases the index date was the date patients became eligible for the study. This could be the date they joined an eligible GP practice or the date the practice became eligible for the study. Prevalent cases will have a previous exposure of childhood maltreatment coded for in their medical records prior to their index date. Thus, prevalent cases can be adults at index date. The index date for unexposed patients was set as the same date as their corresponding exposed patient, to mitigate immortal time bias [38]. The exit date is when follow-up ends for participants. This was set at the earliest date of the following: end date of the study, participant death, outcome occurrence (IMID), patient transferred practice, or last collected data from their general practice. Patients were excluded from the cohort if they had the outcome of interest prior to their index date.

Model covariates used included sex, age at index date, and Townsend deprivation index (quintiles); these have been independently associated with IMID development and hence have been selected as covariates for adjustment [39,40]. The Townsend deprivation quintile is an area-based measure that incorporates information on unemployment, household oversizing, and car or home ownership to measure material deprivation within a population [41]. It is widely used in research [42]; the score is presented as one of five bands, with a higher score representing greater socioeconomic deprivation. For Townsend deprivation quintile, a separate category was used for missing data where exposed patients with missing data were matched to unexposed patients with missing data. The quantity of missing data made this a suitable approach [43].

### 3.4 Statistical analysis

Analysis was conducted using STATA version 17. Categorical data have been described as numbers and proportions whilst continuous data have been presented as means with standard deviation or median with interquartile range (IQR) (for skewed data). Options for handling missing data (such as multiple imputation) were considered, however, after reviewing due to high levels of missingness of key covariates it was deemed more appropriate to include them as a separate missing category in the final analysis [43].

Crude IRs were then calculated (per 100,000 person-years) for each outcome of interest by dividing the number of new outcomes by the total number of person-years at risk. An unadjusted incidence rate ratio (IRR) and adjusted IRR using a Poisson regression are presented. To compare the risk of IMID development in exposed and unexposed groups Cox regression analyses was employed to generate adjusted hazard ratios (aHR). Sex, age at index date, and Townsend deprivation quintile were used to adjust hazard ratios and account for residual confounding following the matching procedure. Finally, sensitivity and subgroup analyses were conducted. An initial sensitivity analysis consisting of the exposed group with only GP-recorded childhood maltreatment codes (excluding those with maltreatment-related concerns) was undertaken. This was then followed by a second sensitivity analysis where incident only cases (excluding prevalent cases) were included in the cohort. A subgroup analysis disaggregated by sex was undertaken to determine if there is any variation in risk of developing IMIDs following GP-recorded childhood maltreatment in males and females.

## 4 Results

**Fig. 1:**
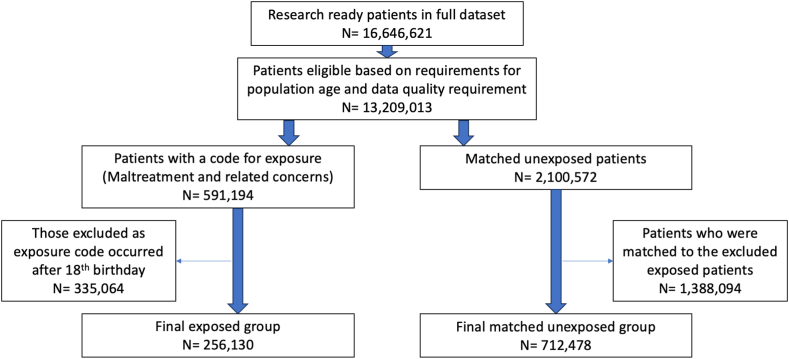
Patient flow diagram.

Following extraction of the dataset, there was a total of 16,646,621 patients present in the eligible practices. A GP had recorded an exposure to childhood maltreatment or related to maltreatment in 591,194 of these patients.These were matched to 2,100,572 patients from the unexposed cohort based on criteria mentioned prior. From this, those who had a practitioner recorded maltreatment exposure after their 18th birthday were excluded, with their respective matched unexposed participant. This resulted in the final study population that was used for analysis, which included 256,130 patients in the exposed group and 712,478 patients, whom they were matched to, in the unexposed group (see Fig. 1).

The exposed group was followed up for a median of 2·46 years (IQR 0·96 - 5·67) compared to 3·46 years (IQR 1·40 - 6·95) in the unexposed group (Table 1). The mean age at cohort entry, sex distribution, and Townsend deprivation quintile were similar across the exposed and unexposed groups due to matching. In the exposed group, a recorded exposure to childhood maltreatment occurred at a mean age of 6·54 years (standard deviation (SD) 5·29). Body mass index (BMI) and smoking had a substantial amount of missing data across both groups. Where smoking status had been recorded, t baseline those exposed to childhood maltreatment were twice as likely to be a current smoker compared to those unexposed (12·70 % vs. 6·82 %).

**Table 1:** Baseline characteristics in those exposed and unexposed to childhood maltreatment.

	Exposed Group	Unexposed Group
**Number of Patients (n)**	256,130	712,478
**Median (IQR) follow-up period (person years)**	2·46 (0·96–5·67)	3·46 (1·40–6·95)
**Mean (SD) age at cohort entry (years)**	12·06 (9·94)	12·12 (10·11)
**Mean age (SD) at exposure (years)**	6·54 (5·29)	n/a
**Sex**		
Male, n (%)	347,912 (48·83)	122,167 (47·70)
Female, n (%)	364,566 (51·17)	133,963 (52·30)
**Body mass index, n (%)**		
Underweight (<18·5 kg/m^2^)	3968 (1·55)	8664 (1·22)
Normal (18·5–24·9 kg/m^2^)	20,278 (7·92)	64,276 (9·02)
Overweight (25·0–30·0 kg/m^2^)	7822 (3·05)	28,052 (3·94)
Obese (>30·0 kg/m^2^)	6478 (2·53)	19,106 (2·68)
Not available	217,584 (84·95)	592,380 (83·14)
**Smoking status, n (%)**		
Current smoker	32,521 (12·70)	48,566 (6·82)
Non-current smoker	39,504 (15·42)	168,375 (23·63)
Not available	184,105 (71·88)	495,537 (69·55)
**Townsend index, n (%)**		
(Least deprived) 1	21,560 (8·42)	69,193 (9·71)
2	25,720 (10·04)	77,424 (10·87)
3	41,908 (16·36)	120,275 (16·88)
4	57,928 (22·59)	155,895 (21·88)
5	58,928 (23·01)	145,239 (20·39)
Not available	50,151 (19·58)	144,452 (20·27)
**Ethnicity, n (%)**		
White	127,046 (49·60)	301,266 (42·28)
Black	6276 (2·45)	20,725 (2·91)
South Asian	4024 (1·57)	23,951 (3.36)
Mixed	5012 (1·96)	10,068 (1·41)
Other	2784 (1·09)	15,951 (2·24)
Missing	110,988 (43·33)	340,517 (47·79)

During the study period, there was a positive effect size for new GP-recorded diagnoses of RhA (aHR 1·39, 95 % CI 1·12 - 1·74) and psoriasis (aHR 1·16, 95 % CI 1·10 - 1·23), when comparing the exposed cohort to the unexposed cohort, with both results being significant. The difference in effect size for MS (aHR 1·07, 95 % CI 0·77 - 1·49) and SLE (aHR 1·28, 95 % CI 0·89 - 1·85) between the exposed group and unexposed group was not significant. By contrast, the risk of IBD and coeliac disease were lower when comparing the exposed cohort to the unexposed, aHR 0·87 (95 % CI 0·75 - 1·00) and 0·74 (95 % CI 0·62 - 0·88) respectively. Additional information is available in Table 2.

**Table 2:** Risk of developing immune-mediate inflammatory disorders in those exposed and unexposed to child maltreatment.

		Number of outcomes	Person-years	Incidence Rate (per 100,000 person years)	Unadjusted Hazard Ratio (95 % CI); p value	Adjusted Hazard Ratio (95 % CI)^a^	P-value for adjusted HR
**IBD**	**Exposed**	236	1,009,120	23·39	0·86 (0·75 - 1·00); 0.046	0·87 (0·75–1·00)	0·056
	**Unexposed**	928	3,380,344	27·45			
**Coeliac Disease**	**Exposed**	151	1,009,555	14·96	0·74 (0·62 - 0·88); 0.001	0·74 (0·62–0·88)	0·001
	**Unexposed**	685	3,381,674	20·26			
**RhA**	**Exposed**	113	1,009,592	11·19	1·46 (1·17 - 1·82); 0.001	1·39 (1·12–1·74)	0·003
	**Unexposed**	263	3,383,376	7·77			
**Psoriasis**	**Exposed**	1533	1,003,335	152·79	1·19 (1·12–1·26); <0.001	1·16 (1·10–1·23)	<0·001
	**Unexposed**	4329	3,362,640	128·74			
**MS**	**Exposed**	48	1,009,927	4·75	1·13 (0·81 - 1·56); 0.474	1·07 (0·77–1·49)	0·673
	**Unexposed**	147	3,383,793	4·34			
**SLE**	**Exposed**	41	1,009,897	4·06	1·38 (0·96 - 1·98); 0.083	1·28 (0·89–1·85)	0·177
	**Unexposed**	101	3,383,882	2·98			

IMIDs (immune-mediated inflammatory disorders); IBD (inflammatory bowel disease); RhA (rheumatoid arthritis); MS (multiple sclerosis); SLE (systemic lupus erythematosus); CI (confidence interval).

a Adjusted for age at index date, sex, and Townsend deprivation quintile at baseline.

In the first sensitivity analysis, following exclusion of those with maltreatment-related concerns and their matched unexposed participant (leaving only those with GP-recorded maltreatment), we found 42,970 (16·78 %) patients remained in the exposed group, who were matched to 126,519 (17·78 %) unexposed patients (appendix S Table 1). At study entry this cohort were older than the main cohort (mean age 18.23 years [SD: 12·49]), with a year shorter median follow-up period (1·58 years [IQR: 1·03–5·85). Distribution of deprivation quintiles and ethnicity were similar to the main cohort. When comparing exposed participants with GP-recorded maltreatment Read codes with those with no record of maltreatment, the effect estimates for new diagnoses of, RhA (aHR: 1·62 [95 % CI: 1·14-2·29]), psoriasis (aHR: 1·32 [95 % CI: 1·17-1·49]), coeliac disease (aHR: 0·59 [95 % CI: 0·37-0·92]), and SLE (aHR: 1·51 [95 % CI: 0·83-2·74]) were slightly higher than those observed in the main analyses. There was a decrease in effect size during follow-up for new diagnoses of MS (aHR: 0·91 [95 % CI: 0·50-1·64]) and IBD (aHR: 0·96 [95 % CI: 0·72-1·28]) in comparison to the main cohort. Additional information is available in appendix S Table 2.

99,711 (38·92 %) patients had achildhood maltreatment Read code recorded during the study period whilst being eligible. This cohort comprised the exposed group for the second sensitivity analysis of incident only cases. They were matched against 256,361 (35·98 %) unexposed patients (appendix S Table 3). The age when maltreatment occurred was similar to the main cohort. The effect size for new diagnoses were: IBD (aHR: 0·69 [95 % CI: 0·51-0·93]), RhA (aHR: 1·23 [95 % CI: 0·70-2·16]), psoriasis (aHR: 1·00 [95 % CI: 0·89-1·12]), SLE (aHR: 1·25 [95 % CI: 0·56-2·77]), coeliac disease (aHR: 0·85 [95 % CI: 0·65-1·11]) and MS (aHR: 1·70 [95 % CI: 0·72-4·00]). Further details can be seen in appendix S Table 4.

The subgroup analysis examined outcomes by sex. 122,167 male patients and 133,963 female patients from the exposed group were matched to 347,912 male patients and 364,569 female patients from the unexposed cohort (appendix S Table 5/6). The mean age for males in the exposed groupat cohort entry was 11·10 (SD 9·51), with mean age when childhood maltreatment occurring at 6·02 years (SD 5·03). This is compared to a mean age at cohort entry of 12·94 years (SD 10·24) for females in the exposed group and mean age when maltreatment occurring at 7·00 years (SD 5·48). There were differences in risk of new diagnoses by sex; for example, RhA (males – aHR: 1·04 [95 % CI: 0·65-1·65]; females – aHR: 1·54 [95 % CI: 1·20-1·99]) and psoriasis (males – aHR: 1·11 [95 % CI: 1·00–1·22]; females – aHR 1·20 [95 % CI: 1·12-1·29]) (appendix S Table 7/8).

## 5 Discussion

As far as we are aware this study represents the first synthesis of data to assess the relationship between childhood maltreatment exposure and the onset of IMIDs using a national UK database. The primary analysis revealed an increased risk of developing RhA and Psoriasis following childhood maltreatment exposure. Subsequent sensitivity analyses included a cohort of only GP-reported cases of childhood maltreatment exposure, which saw the association persist and was more pronounced in females exposed to childhood maltreatment.

Due to the lack of cohort evidence, it is not possible to compare the incidence rates described in our study to the known literature. However, our positive findings for RhA and Psoriasis are largely in line with previous observational data. For example, Wan et al. found 62·3 % of RhA patients had experienced more than one episode of childhood maltreatment, which was higher than the control group [44]. Although, Wan et al. utilised questionnaires and surveys, which could be limited by social desirability and recall bias we demonstrate a similar relationship [44]. Similarly regarding Psoriasis, Simonic et al. evaluated the presence of ACEs in 100 patients with psoriasis using questionnaires, which found ACEs to be more common in those exposed compared to the control group [45]. It is hard to compare results due to differences in methodology, however this study is significant to the literature base by not being hindered by recall bias. We expected RhA to be well-coded as a QoF and thus outcomes better recorded, yet there is also biological rationale supporting an association between the development of RhA and psoriasis following childhood maltreatment. Childhood maltreatment has been shown to dysregulate the hypothalamus-pituitary axis and the immune system, which leads to increased low-grade inflammation, as indicated by elevated levels of interleukin-6 (IL-6) and C-reactive protein (CRP) [46,47]. Studies have shown an elevated level of IL-6 in both RhA and psoriasis patients whilst in IBD there is not a significant elevation of IL-6 [[48], [49], [50]]. Interestingly in contrast to RhA and Psoriasis our study demonstrated a negative association with the subsequent development of coeliac disease. There is limited literature as to why this may be the case but the reduced risk appears somewhat in line with a Swedish study which assessed the development of coeliac disease after childhood stress and found no increase in odds (OR: 0·45 [95 % CI: 0·01–2·65]) [51]. Although we do not anticipate childhood maltreatment to be protective for coeliac disease, this negative association may occur 1) due to socioeconomic inequalities in healthcare access as children who are maltreated are often in lower socioeconomic groups or 2) symptoms such as abdominal pain may be overlooked as physical injuries in this group, and support the need for further research to explore this association [52,53]. Unique to coeliac disease compared to the other IMIDs included is the role of an exogenous exposure to its development. Studies have suggested that the amount and timing of gluten introduction impacts the risk [[54], [55], [56]]. More insight is needed to explain this association. Our non-statistically significant findings for IBD and MS are largely in line with the limited literature in this field [57,58]. Interestingly, a Canadian study found that childhood abuse was associated with ulcerative colitis rather than Crohn's, an analysis we did not undertake here. Regarding SLE where we found a non-significant but positive effect size, Cozier et al. used data from the Black Women's Health Study between 1995 and 2015 and found statistically significant increases in risk of developing SLE following sexual abuse (aHR: 2·51 [95 % CI: 1·29-4·85]) [59]. Although, Cozier et al. defined sexual abuse as their exposure, whereas in our study all forms of childhood maltreatment are considered as the exposure which may explain the difference [59]. the average age for an MS or SLE diagnosis is in the 4th decade of life, therefore this study is limited by its follow up time which could explain the non-significant effect sizes [60,61]. IMIDs are hypothesised to be related diseases. The disparity in effect sizes for childhood maltreatment and developing each IMID raises further questions regarding the nature of their association. It would be expected that the group of IMIDs investigated would have similar effect sizes. This study suggests the pathophysiology of IMIDs, whilst sharing similarities, may have many differences. Despite IMIDs sharing features of immune dysregulation, which can be a consequence of chronic inflammation, each disease has distinct pathophysiological mechanisms. The differences in genetic, cellular and molecular pathways could lead to varying susceptibility to external factors such as childhood maltreatment. Chronic stress, because of childhood maltreatment, can influence the hypothalamic-pituitary-adrenal axis leading to prolonged exposure to stress hormones such as cortisol. Hormonal changes may have varying impact on different IMIDs, which could explain the difference in effect sizes seen in this study. It is important to note the duration of exposure to childhood maltreatment and age of onset could influence the extent of immune dysregulation. The risk of developing IMIDs is multifactorial, the significance of different factors may vary between related IMIDs. This study suggests exposure to childhood maltreatment has a larger role in the risk of developing RhA and Psoriasis, compared to the other IMIDs investigated.

Interestingly, in our subgroup analyses, following childhood maltreatment exposure there was a diferrence in risk of developing IMIDs between sex. The risk of IMIDs was generally higher in the female cohort compared to the male cohort. This has also been seen in the meta-analyses by Wan et al., which for example, concluded emotional abuse was only associated with IMIDs in females and not in males (females OR: 3·11 [95 % CI: 1·18-8·24], males OR: 1·78 [95 % CI: 0·55-5·81]) [44].

Despite the variation in development of IMIDs following childhood maltreatment, a wider public health approach that aims to build resilience in children and the communities of those at risk of maltreatment will help reduce school absences, relieve youth justice systems, and improve the integration of children in education, training, and employment. Clinicians should be made aware of the increased risk of RhA and Psoriasis in those exposed to childhood maltreatment so they can consider early risk-management interventions.Whilst there is a professional and moral obligation to report suspected childhood maltreatment, the lack of evidence surrounding efficacy of interventions has prevented the development of definitive government policy and practice guidance [62]. However, there is an opportunity to promote GP contribution to a public health approach as they are a point of contact for families over a life-course, which builds trusting relationships [63]. GPs are well placed to identify and respond to childhood maltreatment, as a universal service that is located between statutory child protective systems and children or parents/carers [63].

Whilst this study is principle to the evidence base, an updated systematic review is in need to reliably conclude if exposure to childhood maltreatment leads to the progression of IMIDs. Prior to this, primary studies, with similar methods, should be conducted in regions with different healthcare and child protective services, to increase generalisability of these implications to a larger population. As the IMRD-UK database had variation in regional uptake, conducting primary studies in different regions would provide insight where data was more limited. This is important to gain understanding into which forms of abuse are more detrimental, as well as the cultural and genetic factors that impact development of IMIDs. An assessment of the impact of maltreatment frequency will be important to confirm the presence of a dose-response relationship with the development of IMIDs. In addition to this, qualitative research should be undertaken to gain an understanding of the experiences of childhood maltreatment survivors, such as barriers to presentation. Incorporating a collaborative process in study design to allow patient and public involvement will help inform the interpretation of results.

The strengths of this study lie in its use of the IMRD-UK, which is a comprehensive source of population data. A dataset extracted from IMRD-UK allows the results of this study to be generalisable to the UK population, as well as other countries with similar demographic structures, health care systems and child protective services. Further, it helps to mitigate the risk of selection bias and improve internal validity. The structure of data from electronic medical records stored in IMRD-UK has allowed this study to be conducted on a large and characterised cohort. The rigorous design of this study was informed by literature that assessed the use of IMRD-UK in epidemiological research, as well studies exploring other health outcomes following abusive experiences [14,28,[64], [65], [66], [67]].

It is important to be aware of the limitations of this study when considering its results and their significance. The electronic medical records comprising the dataset rely on the accurate inputting of codes by healthcare professionals.As a result, primary care data has a substantial burden of missing data and hidden disease burdens – particularly for our exposure of interest. This may hinder validity due to the propensity of misclassification [68]. The validity of these findings is reliant on the accuracy of coding by GPs in primary care. There is a variety of codes that relate to childhood maltreatment, made evident in this study. It is difficult to ascertain the accuracy of the coding of childhood maltreatment. One study has done work to assess the validity of the coding of childhood maltreatment in primary care [69]. They found that the Read code list used in previous literature, and this study, had a specificity of >97 %. However, it also found the Read code list had a sensitivity of 7.6 % for incident cases and 11.6 % for prevalent cases, probably largely as a result of under-reporting and under-recording. This translates into a large number of cases that are potentially missed using these Read codes. Another limitation of this study is that severe cases of childhood maltreatment may be more likely to be recorded by a GP, as often less severely presenting cases may remain hidden. Further, the recording of IMIDs is not yet validated in primary care data. Ethnicity, smoking and alcohol are both independent risk factors for IMID development and the pro-inflammatory state [25,70]. They were largely missing in the dataset which limited our ability to consider them as confounders in the analysis. Other factors that have been associated with the development of IMIDs and could potentiate confounding include: education status, obesity, presence of comorbidities and family history [[71], [72], [73]]. These were missing from the dataset which meant there could be confounding present which impacts the results of this study [68,74]. Moreover, this study used Townsend deprivation quintiles to account for the effects of socioeconomic deprivation on the development of IMIDs, an area-based measure as opposed to an individual person-based measure. It is also important to consider the median follow-up of the exposed cohort was shorter than the unexposed group. This could be due to children experiencing maltreatment being more likely to move house and thus GP surgery [75]. This could mean the follow-up may not be long enough, considering the effects of childhood maltreatment often present to primary care late and different IMIDs developing over different age groups. Thus, there is a possibility the effects of maltreatment are underestimated in this study. Our sensitivity analysis of incident-only cases showed that all effect sizes for each IMID became insignificant. Incident cases are those where exposure has occurred during the study period. Given the chronicity and relapsing-remitting nature of IMIDs, the relatively short follow-up time results in a right-censoring bias, whereby participants may leave the study before they develop the outcome or receive a recorded diagnosis [76].

In conclusion, our study demonstrated a significant elevated risk of developing some types of IMIDs (RhA and Psoriasis) following exposure to childhood maltreatment. The exact mechanisms behind these associations are unclear and require further research to definitively conclude a relationship. Due to the prevalence of childhood maltreatment and IMIDs in the UK our findings are of public health importance.This retrospective, open cohort study is a key addition to the literature base, which is limited to cross sectional and case-control studies. The various adverse health outcomes associated with childhood maltreatment underscores the importance of a comprehensive public health strategy. The strategy should focus on the prevention and early detection of childhood maltreatment, as well as addressing the risk factors that contribute to the development of IMIDs following childhood maltreatment exposure.

## CRediT authorship contribution statement

**Liam Snook:** Writing – original draft, Investigation, Formal analysis, Data curation. **Sonica Minhas:** Writing – review & editing, Writing – original draft, Formal analysis. **Vrinda Nadda:** Writing – original draft. **Ben Hammond:** Writing – original draft, Formal analysis. **Krishna M. Gokhale:** Software, Resources, Methodology, Investigation. **Julie Taylor:** Writing – review & editing, Supervision. **Caroline Bradbury-Jones:** Writing – review & editing, Supervision. **Siddhartha Bandyopadhyay:** Writing – review & editing, Supervision. **Krishnarajah Nirantharakumar:** Writing – review & editing, Supervision. **Nicola J. Adderley:** Writing – review & editing, Supervision, Methodology, Conceptualization. **Joht Singh Chandan:** Writing – review & editing, Supervision, Resources, Project administration, Methodology, Conceptualization.

## Evidence before this study

Prior to commencing this study, a scoping search to identify gaps in available literature was conducted using Ovid Medline. The search was conducted with the key words “∗childhood maltreatment OR child abuse∗ AND ∗rheumatoid arthritis OR systemic lupus erythematosus OR multiple sclerosis OR psoriasis OR coeliac disease OR inflammatory bowel disease∗” with no age or language restrictions. The search yielded 35 results, which consisted mostly of case-control and cross sectional studies. Whilst these studies largely focused on an individual immune-mediated inflammatory disorder (IMID), findings suggest an association between childhood maltreatment and having an IMID diagnosis. The most recent meta-analysis evaluated the risk of developing some IMIDs following childhood maltreatment and was conducted in 2009. This study included 24 studies (n = 48,801) and provided evidence supporting a positive association between child abuse and IMIDs may exist. However, the conclusions drawn from this review are limited due to the design of included studies and cohort evidence is needed.

**Significance of this research**As far as we are aware, this is the first study of its kind that examines the relationship between primary care recorded childhood maltreatment and the development of IMIDs: rheumatoid arthritis, psoriasis, coeliac disease, multiple sclerosis, inflammatory bowel disease, and systemic lupus erythematosus, on a large-scale in the UK. Our conclusions suggest exposure to childhood maltreatment increases the risk of developing Rheumatoid arthritis and Psoriasis.

## Implications of all available evidence

The findings of this study are pertinent to a substantial proportion of the population due to the prevalence of childhood maltreatment and IMIDs. Our results add to an existing literature base that shows a significant disease burden associated with exposure to childhood maltreatment. It is fundamental to develop public health interventions that aim to improve the identification and primary prevention of childhood maltreatment, as well as improving clinical awareness of knowledge of the associated consequences.

## Ethical approval

Anonymised Data was used from the Data provider to the University of Birmingham. The UK Research Ethics Committee has approved the use of IMRD (reference number: 18/LO/0441); in accordance with this approval, the study protocol for this project (18THIN034) has been reviewed and approved by an independent Scientific Review Committee (SRC).An amendent was made to the protocol for the analysis undertaken in this manuscript. This enabledthe exploration of IMIDs as the previous protocol specified cardiometabolic, mental health and central sensitisation outcomes. IMRD incorporates Data from The Health Improvement Network (THIN), a Cegedim Database. Reference made to THIN is intended to be descriptive of the Data asset licensed by IQVIA. This work has utilised de-identified Data which was provided by patients as part of their routine primary care. Due to the use of de-identifed data there is no opportunity to obtain independent written consent from patients who contribute to the dataset.

## Data availability

Upon publication, the analysis code will be available upon request from the corresponding author (JSC). Data from this study has not been deposited in a publicly available repository as the authors do not have permission to share the licensed data. For access to the raw data that was used for all the analyses, approval must be obtained from the data provider (IQVIA) and independent SRC who approved the ethics for this study who will be able to share the IMRD dataset. It is likely that this process will incur a cost bespoke to the Institution requesting the data. This can be done with support of the corresponding author (JSC).

## Funding

None.

## Role of the funding source

There was no funding source for this study. The final responsibility for the decision to submit this manuscript for publication rested with the corresponding author (JSC), who had full access to all the data in the study.

## Declaration of competing interest

The authors declare that they have no known competing financial interests or personal relationships that could have appeared to influence the work reported in this paper.
