# Supplementary material for: The risk of immune-mediated inflammatory diseases following exposure to childhood maltreatment: A retrospective cohort study using UK primary care data
Source: Heliyon. 2024 Nov 16;10(22):e40493. doi: 10.1016/j.heliyon.2024.e40493 (PMC11617863; doi:10.1016/j.heliyon.2024.e40493)
Supplement: Multimedia component 2 [file mmc2.docx]

Appendices

[Appendix 1: Read Code Lists 2](#_Toc146819232)

[S Table 1: Baseline Characteristics of those exposed and unexposed childhood maltreatment in GP-recorded cases only 12](#_Toc146819233)

[S Table 2: The risk of developing immune-mediated inflammatory disorders in those exposed and unexposed to childhood maltreatment in GP-recorded cases only 13](#_Toc146819234)

[S Table 3: Baseline characteristics in those exposed and unexposed to childhood maltreatment in incident cases only 14](#_Toc146819235)

[S Table 4: The risk of developing immune-mediated inflammatory disorders in those exposed and unexposed to childhood maltreatment in incident cases only 15](#_Toc146819236)

[S Table 5: Baseline characteristics in those exposed and unexposed to childhood maltreatment in males 16](#_Toc146819237)

[S Table 6: Baseline characteristics in those exposed and unexposed to childhood maltreatment in females only 17](#_Toc146819238)

[S Table 7: The risk of developing immune-mediated inflammatory disorders in those exposed and unexposed to childhood maltreatment in males 18](#_Toc146819239)

[S Table 8: The risk of developing immune- mediated inflammatory disorders in those exposed and unexposed to childhood maltreatment in females 19](#_Toc146819240)

# Appendix 1: Read Code Lists

Maltreatment related codes

| Code | Description |
| --- | --- |
| 13IC.00 | Child on at risk register |
| 13ICZ00 | Child on at risk regist NOS |
| 13IM.00 | Child on protection register |
| 13Id.00 | On child protection register |
| 13Iv.00 | Subject to child protection plan |
| 64c..00 | Child protection procedure |
| Z35..00 | Child protection procedure |
| 3874000 | Multidisciplinary case conference |
| 3875000 | Social services case conference |
| 3879000 | Review case conference |
| 8CM6.00 | Child protection plan |
| Z331.00 | Child protection plan |
| 9F2..00 | Child at risk-case conference |
| Z352.00 | Child protection investigation |
| 13VF.00 | At risk violence in the home |
| 13IB.00 | Child in care |
| 13IB000 | Child in foster care |
| 13IB100 | Looked after child |
| 13IV.00 | Looked after child - Children (Scotland) Act 1995 |
| 13ZV.00 | At risk of neglect by others |
| 13ZT.00 | At risk of physical abuse |
| 13ZW.00 | At risk of sexual abuse |
| 13ZR.00 | At risk of emotional/psychological abuse |
| 13VX.00 | At risk of sexual exploitation |
| 13ZZ100 | At risk of psychological abuse |
| 13HP600 | Violence between parents |
| 38C0.00 | Child in care health assessment |
| 6982000 | Fostering medical examination |
| 13If.00 | Child is cause for concern |
| 13Ip.00 | Family is cause for concern |
| 13IF.00 | Child at risk |
| 13IF.11 | Vulnerable child |
| 13IQ.00 | Vulnerable child in family |
| 13IS.00 | Child in need |
| 14XD.00 | History of domestic abuse |
| 14X3.00 | History of domestic violence |
| 13W..11 | Family problems |
| 1BE1.00 | Problem situation |
| 625..00 | A/N care: social risk |
| 625Z.00 | A/N care: social risk NOS |
| 8CM5.00 | Child in need plan |
| 13G4.00 | Social worker involved |
| 8H75.00 | Refer to social worker |
| ZL79.11 | Refer to social worker |
| 8HHB.00 | Referral to Social Services |
| 9NDA.00 | Report received from social services |
| 9N26.00 | Seen by social worker |
| 9Nl6.00 | Seen by social services |
| 9NNV.00 | Under care of social services |
| 9NNk.00 | Under care of social worker |
| 9b0k.00 | Social services report |
| 1J3..00 | Suspected child abuse |
| 1J30.00 | Suspected sexual abuse of child |
| 1J31.00 | Suspected non-accidental injury to child |
| 1J32.00 | Suspected victim of child neglect |

Officially confirmed maltreatment codes

| Code | Description |
| --- | --- |
| 13WT.00 | Child protection observation |
| 13WT000 | Child protection category |
| 13WT100 | Child protection category emotional |
| 13WT200 | Child protection category physical |
| 13WT300 | Child protection category sexual |
| 13WT400 | Child protection category neglect |
| 13W3.00 | Child abuse in family |
| 13W4.00 | Parent/child conflict |
| 13W4000 | Child/parent violence |
| 6254000 | A/N care: H/O child abuse |
| SN55z11 | Child abuse NEC |
| ZV61200 | [V]Child abuse |
| Z352.11 | Child abuse investigation |
| SN55.00 | Child maltreatment syndrome |
| SN55000 | Emotional maltreatment of child |
| SN55011 | Emotional deprivation of child |
| SN55012 | Emotional abuse of child |
| SN55100 | Nutritional maltreatment of child |
| SN55111 | Nutritional deprivation of child |
| SN55112 | Malnutrition in child maltreatment syndrome |
| SN55200 | Non-accidental injury to child |
| SN55211 | NAI - non-accidental injury to child |
| SN55212 | Physical injury to child |
| SN55300 | Battered baby or child syndrome NOS |
| SN55311 | Battered baby syndrome NOS |
| SN55312 | Battered child syndrome NOS |
| SN55400 | Multiple deprivation of child |
| SN55500 | Physical abuse of child |
| SN55600 | Non-accidental traumatic head injury to child |
| SN55z00 | Child maltreatment syndrome NOS |
| SN55z12 | Child deprivation syndrome |
| SN55z13 | Neglect affecting child NEC |
| ZV61213 | [V]Parent - child conflict |
| ZV61212 | [V]Child neglect |
| ZV61211 | [V]Child battering |
| 13II.00 | Child deserted by parents |
| 13II.11 | Child deserted by mother |
| 13Ii.00 | Subject to care order under Children Act 1989 |
| 13Ii000 | Subject to care order under section 20 of Children Act 1989 |
| 13Ii100 | Subject to care order under section 21 of Children Act 1989 |
| 13Ii200 | Subject to care order under section 25 of Children Act 1989 |
| 13Ii300 | Subject to care order under section 31 of Children Act 1989 |
| 13Ij.00 | Subject to interim care order under Children Act 1989 |
| 13Ij000 | Sub to interim care order under section 38 Children Act 1989 |
| 13Ij100 | Emergency protective order section 44 Children Act 1989 |
| 13Ih.00 | Subject to supervision order under Children Act 1989 |
| Z787.00 | Self-neglect |
| 222R.00 | Neglected appearance |
| R037.00 | [D]Insufficient intake of food and water due to self neglect |
| R2y3.11 | [D] Self neglect |
| Ry18.00 | [D]Self neglect |
| SN57000 | Neglect or abandonment |
| TE40.00 | Accidents due to abandonment or neglect of helpless person |
| TLx4.00 | Assault by criminal neglect |
| U3M..00 | [X]Neglect and abandonment |
| U3M0.00 | [X]Neglect and abandonment, by spouse or partner |
| U3M1.00 | [X]Neglect and abandonment, by parent |
| U3M2.00 | [X]Neglect and abandonment, by acquaintance or friend |
| U3My.00 | [X]Neglect and abandonment, by other specified persons |
| U3Mz.00 | [X]Neglect and abandonment, by unspecified person |
| Z787200 | Neglect of clothes |
| Z787400 | Neglect of personal hygiene |
| Z787500 | Neglect of physical health |
| Z787600 | Neglect of dental care |
| Z787700 | Neglect of physical illness |
| Z787800 | Neglect of common dangers |
| ZV1B400 | [V]Personal history of neglect |
| ZV4H300 | [V]Emotional neglect of child |
| ZV4H400 | [V]Other problems related to neglect in upbringing |
| ZVu4B00 | [X]Other problems related to neglect in upbringing |
| 14X6.00 | Victim of sexual abuse |
| 14X..00 | History of abuse |
| 14X0.00 | History of physical abuse |
| 14X1.00 | History of sexual abuse |
| 14X2.00 | History of emotional abuse |
| 14X3.00 | History of domestic violence |
| 14X5.00 | Victim of physical abuse |
| 14X6000 | Victim of sexual harassment |
| 14X7.00 | Victim of emotional abuse |
| 14X8.00 | Victim of domestic violence |
| 14XD.00 | History of domestic abuse |
| 14XD000 | H/O domestic emotional abuse |
| 14XD100 | H/O domestic physical abuse |
| 14XD200 | H/O domestic sexual abuse |
| 14XE.00 | History of being victim of domestic violence |
| 14XF.00 | Victim of human trafficking |
| 14XG.00 | Victim of domestic abuse |
| 14XH.00 | Victim of child sexual exploitation |
| 14XJ.00 | Victim of psychological abuse |
| 14XK.00 | Victim of financial abuse |
| 14XP.00 | Victim of discriminatory abuse |
| 14XR.00 | Victim neglect & acts omission |
| SN57.00 | Maltreatment syndromes |
| SyuH500 | [X]Other maltreatment syndromes |
| TL7..00 | Child battering and other maltreatment |
| TL70.00 | Child battering or other maltreatment by parent |
| TL7y.00 | Child battering or other maltreatment by other spec person |
| TL7z.00 | Child battering or other maltreatment by person NOS |
| U3N..00 | [X]Other maltreatment syndromes |
| U3N0.00 | [X]Other maltreatment syndromes, by spouse or partner |
| U3N1.00 | [X]Other maltreatment syndromes, by parent |
| U3N2.00 | [X]Other maltreatment syndromes, by acquaintance or friend |
| U3N3.00 | [X]Other maltreatment syndromes, by official authorities |
| U3Ny.00 | [X]Other maltreatment syndromes, by other specified persons |
| U3Nz.00 | [X]Other maltreatment syndromes, by unspecified person |
| U3P..00 | [X]Maltreatment |
| U3P0.00 | [X]Maltreatment, by spouse or partner |
| U3P1.00 | [X]Maltreatment, by parent |
| U3P2.00 | [X]Maltreatment, by acquaintance or friend |
| SN42000 | Deprivation of food, unspecified |
| SN43000 | Deprivation of water |
| SN57100 | Sexual abuse |
| SN56000 | Battered person unspecified, syndrome |
| SN57200 | Child affected by Munchausen's by proxy |

Coeliac disease

| MEDICAL_CODE_ID | DESCRIPTION |
| --- | --- |
| 6648 | Coeliac disease monitoring |
| 6648000 | Coeliac disease annual review |
| 8IAp.00 | Coeliac disease annual review declined |
| 9mB..00 | Coeliac disease monitoring invitation |
| 9mB1.00 | Coeliac disease monitoring invitation first letter |
| J690.00 | Coeliac disease |
| J690.11 | Coeliac rickets |
| J690.12 | Gee - Herter disease |
| J690.13 | Gluten enteropathy |
| J690.14 | Sprue - nontropical |
| J690.15 | Steatorrhea - idiopathic |
| J690000 | Congenital coeliac disease |
| J690100 | Acquired coeliac disease |
| J690z00 | Coeliac disease NOS |
| ZC2C200 | Dietary advice for coeliac disease |

Psoriasis

| MEDICAL_CODE_ID | DESCRIPTION |
| --- | --- |
| 14F2.00 | H/O: psoriasis |
| M16..00 | Psoriasis and similar disorders |
| M160.00 | Psoriatic arthropathy |
| M160.11 | Psoriatic arthritis |
| M160000 | Psoriasis spondylitica |
| M160100 | Distal interphalangeal psoriatic arthropathy |
| M160200 | Arthritis mutilans |
| M160z00 | Psoriatic arthropathy NOS |
| M161.00 | Other psoriasis |
| M161000 | Psoriasis unspecified |
| M161100 | Psoriasis annularis |
| M161200 | Psoriasis circinata |
| M161300 | Psoriasis diffusa |
| M161400 | Psoriasis discoidea |
| M161500 | Psoriasis geographica |
| M161600 | Guttate psoriasis |
| M161700 | Psoriasis gyrata |
| M161800 | Psoriasis inveterata |
| M161900 | Psoriasis ostracea |
| M161A00 | Psoriasis palmaris |
| M161B00 | Psoriasis plantaris |
| M161C00 | Psoriasis punctata |
| M161D00 | Pustular psoriasis |
| M161E00 | Psoriasis universalis |
| M161F00 | Psoriasis vulgaris |
| M161F11 | Chronic large plaque psoriasis |
| M161H00 | Erythrodermic psoriasis |
| M161J00 | Flexural psoriasis |
| M161z00 | Psoriasis NOS |
| M166.00 | Palmoplantar pustular psoriasis |
| M16y.00 | Other psoriasis and similar disorders |
| M16y000 | Scalp psoriasis |
| M16z.00 | Psoriasis and similar disorders NOS |
| Myu3000 | [X]Other psoriasis |
| N045200 | Juvenile arthritis in psoriasis |
| Nyu1300 | [X]Other psoriatic arthropathies |

Rheumatoid Arthritis

| MEDICAL_CODE_ID | DESCRIPTION |
| --- | --- |
| 14G1.00 | H/O: rheumatoid arthritis |
| 2G25.00 | O/E - hands - ulnar deviation |
| 2G25.11 | O/E - ulnar deviation |
| 2G27.00 | O/E-hands-rheumatoid spindling |
| 66H..13 | Rheumatoid arthrit. monitoring |
| 7P20300 | Delivery of rehabilitation for rheumatoid arthritis |
| 9mM..00 | Rheumatoid arthritis monitoring invitation |
| 9mM0.00 | Rheumatoid arthritis monitoring invitation first letter |
| 9mM1.00 | Rheumatoid arthritis monitoring invitation second letter |
| 9mM2.00 | Rheumatoid arthritis monitoring invitation third letter |
| 9mM3.00 | Rheumatoid arthritis monitoring verbal invitation |
| 9mM4.00 | Rheumatoid arthritis monitoring telephone invitation |
| F371200 | Polyneuropathy in rheumatoid arthritis |
| F396400 | Myopathy due to rheumatoid arthritis |
| G5y8.00 | Rheumatoid myocarditis |
| G5yA.00 | Rheumatoid carditis |
| H570.00 | Rheumatoid lung |
| N005.00 | Adult Still's Disease |
| N040T00 | Flare of rheumatoid arthritis |
| N04..00 | Rheumatoid arthritis and other inflammatory polyarthropathy |
| N040.00 | Rheumatoid arthritis |
| N040000 | Rheumatoid arthritis of cervical spine |
| N040100 | Other rheumatoid arthritis of spine |
| N040200 | Rheumatoid arthritis of shoulder |
| N040300 | Rheumatoid arthritis of sternoclavicular joint |
| N040400 | Rheumatoid arthritis of acromioclavicular joint |
| N040500 | Rheumatoid arthritis of elbow |
| N040600 | Rheumatoid arthritis of distal radio-ulnar joint |
| N040700 | Rheumatoid arthritis of wrist |
| N040800 | Rheumatoid arthritis of MCP joint |
| N040900 | Rheumatoid arthritis of PIP joint of finger |
| N040A00 | Rheumatoid arthritis of DIP joint of finger |
| N040B00 | Rheumatoid arthritis of hip |
| N040C00 | Rheumatoid arthritis of sacro-iliac joint |
| N040D00 | Rheumatoid arthritis of knee |
| N040E00 | Rheumatoid arthritis of tibio-fibular joint |
| N040F00 | Rheumatoid arthritis of ankle |
| N040G00 | Rheumatoid arthritis of subtalar joint |
| N040H00 | Rheumatoid arthritis of talonavicular joint |
| N040J00 | Rheumatoid arthritis of other tarsal joint |
| N040K00 | Rheumatoid arthritis of 1st MTP joint |
| N040L00 | Rheumatoid arthritis of lesser MTP joint |
| N040M00 | Rheumatoid arthritis of IP joint of toe |
| N040N00 | Rheumatoid vasculitis |
| N040P00 | Seronegative rheumatoid arthritis |
| N040Q00 | Rheumatoid bursitis |
| N040R00 | Rheumatoid nodule |
| N040S00 | Rheumatoid arthritis - multiple joint |
| N041.00 | Felty's syndrome |
| N042.00 | Other rheumatoid arthropathy + visceral/systemic involvement |
| N042100 | Rheumatoid lung disease |
| N042200 | Rheumatoid nodule |
| N042z00 | Rheumatoid arthropathy + visceral/systemic involvement NOS |
| N047.00 | Seropositive errosive rheumatoid arthritis |
| N04X.00 | Seropositive rheumatoid arthritis, unspecified |
| N04y000 | Rheumatoid lung |
| N04y011 | Caplan's syndrome |
| N04y012 | Fibrosing alveolitis associated with rheumatoid arthritis |
| N04y200 | Adult-onset Still's disease |
| N362200 | Swan-neck finger deformity |
| Nyu1000 | [X]Rheumatoid arthritis+involvement/other organs or systems |
| Nyu1100 | [X]Other seropositive rheumatoid arthritis |
| Nyu1200 | [X]Other specified rheumatoid arthritis |
| Nyu1G00 | [X]Seropositive rheumatoid arthritis, unspecified |

Systemic Lupus Erythematous

| MEDICAL_CODE_ID | DESCRIPTION |
| --- | --- |
| F371000 | Polyneuropathy in disseminated lupus erythematosus |
| F396100 | Myopathy due to disseminated lupus erythematosus |
| F4D3300 | Eyelid discoid lupus erythematosus |
| H57y400 | Lung disease with systemic lupus erythematosus |
| K01x400 | Nephrotic syndrome in systemic lupus erythematosus |
| K01x411 | Lupus nephritis |
| K0B4000 | Renal tubulo-interstitial disorder in SLE |
| M154.00 | Lupus erythematosus |
| M154000 | Lupus erythematosus chronicus |
| M154100 | Discoid lupus erythematosus |
| M154200 | Lupus erythematosus migrans |
| M154300 | Lupus erythematosus nodularis |
| M154400 | Lupus erythematosus profundus |
| M154500 | Lupus erythematosus tumidus |
| M154600 | Lupus erythematosus unguium mutilans |
| M154700 | Subacute cutaneous lupus erythematosus |
| M154z00 | Lupus erythematosus NOS |
| Myu7800 | [X]Other local lupus erythematosus |
| N000.00 | Systemic lupus erythematosus |
| N000000 | Disseminated lupus erythematosus |
| N000100 | Libman-Sacks disease |
| N000200 | Drug-induced systemic lupus erythematosus |
| N000300 | Systemic lupus erythematosus with organ or sys involv |
| N000400 | Systemic lupus erythematosus with pericarditis |
| N000600 | Cerebral lupus |
| N000z00 | Systemic lupus erythematosus NOS |
| Nyu4300 | [X]Other forms of systemic lupus erythematosus |
| ZRq8.00 | Systemic lupus activity measure |
| ZRq8.11 | SLAM - Systemic lupus activity measure |
| ZRq9.00 | Systemic lupus erythematosus disease activity index |
| ZRq9.11 | SLEDAI-Sys lup ery dis act ind |

Inflammatory Bowel Disease

| MEDICAL_CODE_ID | DESCRIPTION |
| --- | --- |
| J4...12 | Inflammatory bowel disease |
| J40..00 | Regional enteritis - Crohn's disease |
| J40..11 | Crohn's disease |
| J400200 | Crohn's disease of the terminal ileum |
| J400300 | Crohn's disease of the ileum unspecified |
| J400400 | Crohn's disease of the ileum NOS |
| J400500 | Exacerbation of Crohn's disease of small intestine |
| J400z00 | Crohn's disease of the small bowel NOS |
| J401200 | Exacerbation of Crohn's disease of large intestine |
| J401z00 | Crohn's disease of the large bowel NOS |
| J401z11 | Crohn's colitis |
| J40z.11 | Crohn's disease NOS |
| J41..12 | Ulcerative colitis and/or proctitis |
| J410.00 | Ulcerative proctocolitis |
| J410000 | Ulcerative ileocolitis |
| J410100 | Ulcerative colitis |
| J410200 | Ulcerative rectosigmoiditis |
| J410300 | Ulcerative proctitis |
| J410400 | Exacerbation of ulcerative colitis |
| J410z00 | Ulcerative proctocolitis NOS |
| J411.00 | Ulcerative (chronic) enterocolitis |
| J412.00 | Ulcerative (chronic) ileocolitis |
| J413.00 | Ulcerative pancolitis |
| J41y.00 | Other idiopathic proctocolitis |
| Jyu4000 | [X]Other Crohn's disease |
| Jyu4100 | [X]Other ulcerative colitis |
| N031000 | Arthropathy in ulcerative colitis |
| N031100 | Arthropathy in Crohn's disease |
| N045300 | Juvenile arthritis in Crohn's disease |
| N045400 | Juvenile arthritis in ulcerative colitis |

Multiple Sclerosis

| MEDICAL_CODE_ID | DESCRIPTION |
| --- | --- |
| 666A.00 | Multiple sclerosis review |
| 666B.00 | Multiple sclerosis multidisciplinary review |
| 8Cc0.00 | Management of multiple sclerosis in onset phase |
| 8Cc1.00 | Management of multiple sclerosis in early disease phase |
| 8Cc2.00 | Management of multiple sclerosis in stable disability phase |
| 8Cc4.00 | Management of multiple sclerosis in palliative phase |
| 8CS1.00 | Multiple sclerosis care plan agreed |
| 8IAb.00 | Multiple sclerosis review declined |
| 9kG..00 | Spec serv for pat with multiple sclerosis - enh serv admin |
| 9mD..00 | Multiple sclerosis monitoring administration |
| 9mD0.00 | Multiple sclerosis monitoring first letter |
| 9mD1.00 | Multiple sclerosis monitoring second letter |
| F20..00 | Multiple sclerosis |
| F20..11 | Disseminated sclerosis |
| F200.00 | Multiple sclerosis of the brain stem |
| F201.00 | Multiple sclerosis of the spinal cord |
| F202.00 | Generalised multiple sclerosis |
| F203.00 | Exacerbation of multiple sclerosis |
| F204.00 | Benign multiple sclerosis |
| F206.00 | Primary progressive multiple sclerosis |
| F207.00 | Relapsing and remitting multiple sclerosis |
| F208.00 | Secondary progressive multiple sclerosis |
| F20z.00 | Multiple sclerosis NOS |

# S Table 1: Baseline Characteristics of those exposed and unexposed childhood maltreatment in GP-recorded cases only

|  | **Exposed Group** | **Unexposed Group** |
| --- | --- | --- |
| **Number of Patients (n)** | 42,970 | 126,518 |
| **Median (IQR) follow-up period (person years)** | 2·58 (1·03 – 5·85) | 3·45 (1·43 – 6·94) |
| **Mean (SD) age at cohort entry (years)** | 18·24 (12·50) | 18·16 (12·79) |
| **Mean age (SD) at exposure (years)** | 7·69 (5·20) | N/A |
| **Sex** |  |  |
| Male, n (%) | 16,604 (38·64) | 50,798 (40·15) |
| Female, n (%) | 26,366 (61·36) | 75,720 (59·85) |
| **Body mass index, n (%)** |  |  |
| Underweight (<18·5 kg/m^2^) | 915 (2·13) | 2,348 (1·86) |
| Normal (18·5-24·9 kg/m^2^) | 5,766 (13·42) | 22,022 (17·41) |
| Overweight (25·0-30·0 kg/m^2^) | 2,583 (6·01) | 10,553 (8·34) |
| Obese (>30·0 kg/m^2^) | 2,402 (5·59) | 7,522 (5·95) |
| Not available | 31,304 (72·85) | 84,073 (66·45) |
| **Smoking status, n (%)** |  |  |
| Current smoker | 9,549 (22·22) | 16,334 (12·91) |
| Non-current smoker | 8,585 (19·98) | 45,983 (36·35) |
| Not available | 24,836 (57·80) | 64,201 (50·74) |
| **Townsend index, n (%)** |  |  |
| (Least deprived) 1 | 3,626 (8·44) | 12,256 (9·69) |
| 2 | 4,510 (10·50) | 74,078 (11·13) |
| 3 | 7,128 (16·59) | 21,266 (16·81) |
| 4 | 9,960 (23·18) | 27,849 (22·01) |
| 5 | 10,055 (23·40) | 26,198 (20·71) |
| Not available | 7,691 (17·90) | 24,871 (19·66) |
| **Ethnicity, n (%)** |  |  |
| White | 22,655 (52·72) | 52,784 (41·72) |
| Black | 823 (1·92) | 3,172 (2·51) |
| South Asian | 727 (1·69) | 4,891 (3·87) |
| Mixed | 731 (1·70) | 1,606 (1·27) |
| Other | 355 (0·83) | 3,014 (2·38) |
| Missing | 17,679 (41·14) | 61,051 (48·25) |

# S Table 2: The risk of developing immune-mediated inflammatory disorders in those exposed and unexposed to childhood maltreatment in GP-recorded cases only

|  |  | **Number of outcomes** | **Person-years** | **Incidence Rate (per 100,000 person years)** | **Unadjusted Hazard Ratio (95% CI)** | **Adjusted Hazard Ratio (95% CI)*** | **P-value for adjusted HR** |
| --- | --- | --- | --- | --- | --- | --- | --- |
| **IBD** | **Exposed** | 60 | 177,303 | 33·84 | 0·96 (0·72 - 1·28); 0.796 | 0·96 (0·72 – 1·28) | 0·799 |
|  | **Unexposed** | 215 | 610,509 | 35·22 |  |  |  |
|  |  |  |  |  |  |  |  |
| **Coeliac Disease** | **Exposed** | 22 | 177,459 | 12·40 | 0·60 (0·38 - 0·94); 0.027 | 0·59 (0·37 – 0·92) | 0·022 |
|  | **Unexposed** | 125 | 611,061 | 20·46 |  |  |  |
|  |  |  |  |  |  |  |  |
| **RhA** | **Exposed** | 47 | 177,285 | 26·51 | 1·68 (1·19 - 2·39); 0.003 | 1·62 (1·14 – 2·29) | 0·007 |
|  | **Unexposed** | 96 | 611,170 | 15·71 |  |  |  |
|  |  |  |  |  |  |  |  |
| **Psoriasis** | **Exposed** | 360 | 175,830 | 204·74 | 1·35 (1·19 - 1·53); <0.001 | 1·32 (1·17 – 1·49) | <0·001 |
|  | **Unexposed** | 915 | 606,882 | 150·77 |  |  |  |
|  |  |  |  |  |  |  |  |
| **MS** | **Exposed** | 14 | 177,490 | 7·89 | 0·96 (0·53 - 1·74); 0.904 | 0·91 (0·50 – 1·64) | 0·749 |
|  | **Unexposed** | 51 | 611,376 | 8·34 |  |  |  |
|  |  |  |  |  |  |  |  |
| **SLE** | **Exposed** | 16 | 177,418 | 9·02 | 1·65 (0·91 - 2·99); 0.099 | 1·51 (0·83 – 2·74) | 0·176 |
|  | **Unexposed** | 34 | 611,412 | 5·56 |  |  |  |

IMIDs (immune-mediated inflammatory disorders); IBD (inflammatory bowel disease); RhA (rheumatoid arthritis); MS (multiple sclerosis); SLE (systemic lupus erythematosus); CI (confidence interval)

*Adjusted for age at index date, sex, and Townsend deprivation quintile at baseline

# S Table 3: Baseline characteristics in those exposed and unexposed to childhood maltreatment in incident cases only

|  | **Exposed Group** | **Unexposed Group** |
| --- | --- | --- |
| **Number of Patients (n)** | 99,711 | 256,360 |
| **Median (IQR) follow-up period (person years)** | 2·69 (0·99 – 5·97) | 3·29 (1·31 – 6·57) |
| **Mean (SD) age at cohort entry (years)** | 6·66 (5·31) | 6·48 (5·01) |
| **Mean age (SD) at exposure (years)** | 6·66 (5·31) |  |
| **Sex** |  |  |
| Male, n (%) | 50,084 (50·23) | 129,562 (50·54) |
| Female, n (%) | 49,627 (49·77) | 126,798 (49·46) |
| **Body mass index, n (%)** |  |  |
| Underweight (<18·5 kg/m^2^) | 499 (0·50) | 563 (0·22) |
| Normal (18·5-24·9 kg/m^2^) | 1,604 (1·61) | 1,171 (0·46) |
| Overweight (25·0-30·0 kg/m^2^) | 355 (0·36) | 268 (0·10) |
| Obese (>30·0 kg/m^2^) | 231 (0·23) | 179 (0·07) |
| Not available | 97,022 (97·30) | 254,179 (99·15) |
| **Smoking status, n (%)** |  |  |
| Current smoker | 2,112 (2·12) | 816 (0·32) |
| Non-current smoker | 10,535 (10·57) | 23,704 (9·25) |
| Not available | 87,064 (87·32) | 231,840 (90·44) |
| **Townsend index, n (%)** |  |  |
| (Least deprived) 1 | 8106 (8·13) | 24445 (9·54) |
| 2 | 9068 (9·09) | 25564 (9·97) |
| 3 | 15807 (15·85) | 42295 (16·50) |
| 4 | 21945 (22·01) | 55162 (21·52) |
| 5 | 23279 (23·35) | 54447 (21·24) |
| Not available | 21506 (21·57) | 54447 (21·24) |
| **Ethnicity, n (%)** |  |  |
| White | 41655 (41·78) | 106914 (41·70) |
| Black | 3037 (3·05) | 9009 (3·51) |
| South Asian | 2276 (2·28) | 9982 (3·89) |
| Mixed | 2000 (2·01) | 4412 (1·72) |
| Other | 1474 (1·48) | 5940 (2·32) |
| Missing | 49269 (49·41) | 120103 (46·85) |

# S Table 4: The risk of developing immune-mediated inflammatory disorders in those exposed and unexposed to childhood maltreatment in incident cases only

|  | |  | **Number of outcomes** | **Person-years** | **Incidence Rate (per 100,000 person years)** | **Unadjusted Hazard Ratio (95% CI); p value** | **Adjusted Hazard Ratio (95% CI)*** | **P-value for adjusted HR** |
| --- | --- | --- | --- | --- | --- | --- | --- | --- |
| **IBD** | | **Exposed** | 54 | 403,533 | 13·38 | 0·74 (0·55 - 0·99); 0.044 | 0·69 (0·51 – 0·93) | 0·016 |
|  |  | **Unexposed** | 209 | 1,143,674 | 18·27 |  |  |  |
|  |  |  |  |  |  |  |  |  |
| **Coeliac Disease** | | **Exposed** | 68 | 403,486 | 16·85 | 0·82 (0·63 - 1·07); 0.151 | 0·85 (0·65 – 1·11) | 0·226 |
|  |  | **Unexposed** | 235 | 1,143,573 | 20·55 |  |  |  |
|  |  |  |  |  |  |  |  |  |
| **RhA** | | **Exposed** | 18 | 403,676 | 4·46 | 1·32 (0·76 - 2·31); 0.327 | 1·23 (0·70 – 2·16) | 0·471 |
|  |  | **Unexposed** | 39 | 1,144,312 | 3·41 |  |  |  |
|  |  |  |  |  |  |  |  |  |
| **Psoriasis** | | **Exposed** | 433 | 401,801 | 107·76 | 1·05 (0·95 - 1·18); 0.339 | 1·00 (0·89 – 1·12) | 0·978 |
|  |  | **Unexposed** | 1167 | 1,138,949 | 102·46 |  |  |  |
|  |  |  |  |  |  |  |  |  |
| **MS** | | **Exposed** | 9 | 403,714 | 2·23 | 1·98 (0·85 - 4·63); 0.116 | 1·70 (0·72 – 4·00) | 0·228 |
|  |  | **Unexposed** | 13 | 1,114,429 | 1·14 |  |  |  |
|  |  |  |  |  |  |  |  |  |
| **SLE** | | **Exposed** | 9 | 403,716 | 2·23 | 1·37 (0·62 - 3·02); 0.440 | 1·25 (0·56 – 2·77) | 0·585 |
|  |  | **Unexposed** | 19 | 1,114,368 | 1·66 |  |  |  |

IMIDs (immune-mediated inflammatory disorders); IBD (inflammatory bowel disease); RhA (rheumatoid arthritis); MS (multiple sclerosis); SLE (systemic lupus erythematosus); CI (confidence interval)

*Adjusted for age at index date, sex, and Townsend deprivation quintile at baseline

# S Table 5: Baseline characteristics in those exposed and unexposed to childhood maltreatment in males

|  | **Exposed** | **Unexposed** |
| --- | --- | --- |
| **Number of patients** | 122,167 | 347,912 |
| **Median (IQR) follow-up period (person years)** | 2·52 (1·00 – 5·74) | 1·48 (0·57 – 3·63) |
| **Mean (SD) age at cohort entry (years)** | 11·10 (9·51) | 11·38 (9·76) |
| **Mean (SD) age at which maltreatment occurred (years)** | 6·02 (5·03) | n/a |
| **Body mass index, n (%)** |  |  |
| Underweight (<18·5 kg/m^2^) | 1643 (1·34) | 3137 (0·90) |
| Normal (18·5-24·9 kg/m^2^) | 7838 (6·42) | 22624 (6·50) |
| Overweight (25·0-29·9 kg/m^2^) | 2774 (2·27) | 10967 (3·15) |
| Obese (>30·0 kg/m^2^) | 1663 (1·36) | 5569 (1·60) |
| Not available | 108249 (88·61) | 305615 (87·84) |
| **Smoking status, n (%)** |  |  |
| Current smoker | 12839 (10·51) | 21831 (6·27) |
| Non-current smoker | 16073 (13·16) | 69269 (19·91) |
| Not available | 93255 (76·33) | 256812 (73·82) |
| **Townsend index, n (%)** |  |  |
| (Least deprived) 1 | 10175 (8·33) | 33097 (9·51) |
| 2 | 12288 (10·06) | 37565 (10·80) |
| 3 | 19877 (16·27) | 58142 (16·71) |
| 4 | 27644 (22·63) | 76612 (22·02) |
| 5 | 27828 (22·78) | 71177 (20·46) |
| Not available | 24355 (19·94) | 71319 (20·50) |
| **Ethnicity, n (%)** |  |  |
| White | 59022 (48·31) | 143375 (41·21) |
| Black | 3112 (2·55) | 9969 (2·87) |
| South Asian | 1966 (1·61) | 11970 (3·44) |
| Mixed | 2439 (2·00) | 5039 (1·45) |
| Other | 1497 (1·23) | 7628 (2·19) |
| Missing | 54131 (44·31) | 169931 (48·84) |

# S Table 6: Baseline characteristics in those exposed and unexposed to childhood maltreatment in females only

|  | **Exposed** | **Unexposed** |
| --- | --- | --- |
| **Number of patients** | 133963 | 364566 |
| **Median (IQR) follow-up period (person years)** | 2·40 (0·94 – 5·60) | 3·31 (1·33 – 6·70) |
| **Mean (SD) age at cohort entry (years)** | 12·94 (10·24) | 12·81 (10·38) |
| **Mean (SD) age at which maltreatment occurred (years)** | 7·00 (5·48) | n/a |
| **Body mass index, n (%)** |  |  |
| Underweight (<18·5 kg/m^2^) | 2325 (1·74) | 5527 (1·52) |
| Normal (18·5-24·9 kg/m^2^) | 12440 (9·29) | 41652 (11·43) |
| Overweight (25·0-29·9 kg/m^2^) | 5048 (3·77) | 17085 (4·69) |
| Obese (>30·0 kg/m^2^) | 4815 (3·59) | 13537 (3·71) |
| Not available | 109335 (81·62) | 286765 (78·66) |
| **Smoking status, n (%)** |  |  |
| Current smoker | 19682 (14·69) | 26735 (7·33) |
| Non-current smoker | 23431 (17·49) | 99106 (27·18) |
| Not available | 90850 (67·82) | 238725 (65·48) |
| **Townsend index, n (%)** |  |  |
| (Least deprived) 1 | 11385 (8·50) | 36096 (9·90) |
| 2 | 13432 (10·03) | 39859 (10·93) |
| 3 | 22031 (16·45) | 62133 (17·04) |
| 4 | 30219 (22·56) | 79283 (21·75) |
| 5 | 31100 (23·22) | 74062 (20·32) |
| Not available | 25796 (19·26) | 73133 (20·06) |
| **Ethnicity, n (%)** |  |  |
| White | 68024 (50·78) | 157891 (43·31) |
| Black | 3164 (2·36) | 10756 (2·95) |
| South Asian | 2058 (1·54) | 11981 (3·29) |
| Mixed | 2573 (1·92) | 5029 (1·38) |
| Other | 1287 (0·96) | 8323 (2·28) |
| Missing | 56857 (42·44) | 170586 (46·79) |

# S Table 7: The risk of developing immune-mediated inflammatory disorders in those exposed and unexposed to childhood maltreatment in males

|  |  | **Number of outcomes** | **Person-years** | **Incidence Rate (per 100,000 person years)** | **Unadjusted Hazard Ratio (95% CI); p value** | **Adjusted Hazard Ratio (95% CI)*** | **P-value for adjusted HR** |
| --- | --- | --- | --- | --- | --- | --- | --- |
| **IBD** | **Exposed** | 104 | 486,124 | 21·39 | 0·75 (0·61 - 0·92); 0.007 | 0·76 (0·62 – 0·95) | 0·013 |
|  | **Unexposed** | 499 | 1,707,154 | 29·23 |  |  |  |
|  |  |  |  |  |  |  |  |
| **Coeliac Disease** | **Exposed** | 42 | 486,408 | 8·63 | 0·66 (0·48 - 0·92); 0.014 | 0·67 (0·48 – 0·93) | 0·016 |
|  | **Unexposed** | 221 | 1,708,452 | 12·94 |  |  |  |
|  |  |  |  |  |  |  |  |
| **RhA** | **Exposed** | 23 | 486,460 | 4·73 | 1·02 (0·64 - 1·62); 0.931 | 1·04 (0·65 – 1·65) | 0·883 |
|  | **Unexposed** | 81 | 1,709,018 | 4·74 |  |  |  |
|  |  |  |  |  |  |  |  |
| **Psoriasis** | **Exposed** | 533 | 484,088 | 110·10 | 1·10 (1·00 - 1·21); 0.060 | 1·11 (1·00 – 1·22) | 0·039 |
|  | **Unexposed** | 1731 | 1,700,581 | 101·79 |  |  |  |
|  |  |  |  |  |  |  |  |
| **MS** | **Exposed** | 15 | 486,521 | 3·08 | 1·80 (0·97 - 3·33); 0.063 | 1·88 (1·02 – 3·49) | 0·045 |
|  | **Unexposed** | 31 | 1,709,181 | 1·81 |  |  |  |
|  |  |  |  |  |  |  |  |
| **SLE** | **Exposed** | 3 | 486,554 | 0·62 | 0·56 (0·17 - 1·89); 0.351 | 0·58 (0·17 – 1·95) | 0·378 |
|  | **Unexposed** | 20 | 1,709,226 | 1·17 |  |  |  |

IMIDs (immune-mediated inflammatory disorders); IBD (inflammatory bowel disease); RhA (rheumatoid arthritis); MS (multiple sclerosis); SLE (systemic lupus erythematosus); CI (confidence interval)

*Adjusted for age at index date, sex, and Townsend deprivation quintile at baseline

# S Table 8: The risk of developing immune- mediated inflammatory disorders in those exposed and unexposed to childhood maltreatment in females

|  |  | **Number of outcomes** | **Person-years** | **Incidence Rate (per 100,000 person years)** | **Unadjusted Hazard Ratio (95% CI); p value** | **Adjusted Hazard Ratio (95% CI)*** | **P-value for adjusted HR** |
| --- | --- | --- | --- | --- | --- | --- | --- |
| **IBD** | **Exposed** | 132 | 522996 | 25·24 | 0·99 (0·91 – 1·20); 0.916 | 0·97 (0·80 – 1·19) | 0·799 |
|  | **Unexposed** | 429 | 1673190 | 25·64 |  |  |  |
|  |  |  |  |  |  |  |  |
| **Coeliac Disease** | **Exposed** | 109 | 523147 | 20·84 | 0·75 (0·61 - 0·93); 0.008 | 0·77 (0·62 – 0·94) | 0·012 |
|  | **Unexposed** | 464 | 1673223 | 27·73 |  |  |  |
|  |  |  |  |  |  |  |  |
| **RhA** | **Exposed** | 90 | 523133 | 17·20 | 1·58 (1·23 - 2·04); <0.001 | 1·54 (1·20 – 1·99) | 0·001 |
|  | **Unexposed** | 182 | 1674357 | 10·87 |  |  |  |
|  |  |  |  |  |  |  |  |
| **Psoriasis** | **Exposed** | 1000 | 519246 | 192·59 | 1·23 (1·14 - 1·32); <0.001 | 1·20 (1·12 – 1·29) | <0·001 |
|  | **Unexposed** | 2598 | 1662058 | 156·31 |  |  |  |
|  |  |  |  |  |  |  |  |
| **MS** | **Exposed** | 33 | 523406 | 3·08 | 0·93 (0·63 - 1·37); 0.704 | 0·89 (0·61 – 1·32) | 0·572 |
|  | **Unexposed** | 116 | 1674612 | 1·81 |  |  |  |
|  |  |  |  |  |  |  |  |
| **SLE** | **Exposed** | 38 | 523343 | 7·26 | 1·51 (1·03 - 2·22); 0.037 | 1·44 (0·98 – 2·12) | 0·065 |
|  | **Unexposed** | 81 | 1674655 | 4·84 |  |  |  |

IMIDs (immune-mediated inflammatory disorders); IBD (inflammatory bowel disease); RhA (rheumatoid arthritis); MS (multiple sclerosis); SLE (systemic lupus erythematosus); CI (confidence interval)

*Adjusted for age at index date, sex, and Townsend deprivation quintile at baseline
